# Evaluating impacts using a BACI design, ratios, and a Bayesian approach with a focus on restoration

**DOI:** 10.1007/s10661-016-5526-6

**Published:** 2016-09-08

**Authors:** Mary M. Conner, W. Carl Saunders, Nicolaas Bouwes, Chris Jordan

**Affiliations:** 1 Department of Wildland Resources, Utah State University, 5230 Old Main Hill, Logan, UT 84322 USA; 2 Department of Watershed Sciences, Utah State University, 5210 Old Main Hill, Logan, UT 84322 USA; 3 Eco Logical Research, Inc., Box 706, Providence, UT 84332 USA; 4 NOAA Fisheries, Northwest Fisheries Science Center, Mathematical Ecology and Systems Monitoring Program, 2725 Montlake Blvd E, Seattle, WA 98112 USA

**Keywords:** Bayesian approach, BACI, Hierarchical model, MCMC, *Oncorhynchus mykiss*, Restoration impact, Steelhead

## Abstract

Before-after-control-impact (BACI) designs are an effective method to evaluate natural and human-induced perturbations on ecological variables when treatment sites cannot be randomly chosen. While effect sizes of interest can be tested with frequentist methods, using Bayesian Markov chain Monte Carlo (MCMC) sampling methods, probabilities of effect sizes, such as a ≥20 % increase in density after restoration, can be directly estimated. Although BACI and Bayesian methods are used widely for assessing natural and human-induced impacts for field experiments, the application of hierarchal Bayesian modeling with MCMC sampling to BACI designs is less common. Here, we combine these approaches and extend the typical presentation of results with an easy to interpret ratio, which provides an answer to the main study question—“How much impact did a management action or natural perturbation have?” As an example of this approach, we evaluate the impact of a restoration project, which implemented beaver dam analogs, on survival and density of juvenile steelhead. Results indicated the probabilities of a ≥30 % increase were high for survival and density after the dams were installed, 0.88 and 0.99, respectively, while probabilities for a higher increase of ≥50 % were variable, 0.17 and 0.82, respectively. This approach demonstrates a useful extension of Bayesian methods that can easily be generalized to other study designs from simple (e.g., single factor ANOVA, paired *t* test) to more complicated block designs (e.g., crossover, split-plot). This approach is valuable for estimating the probabilities of restoration impacts or other management actions.

**Electronic supplementary material:**

The online version of this article (doi:10.1007/s10661-016-5526-6) contains supplementary material, which is available to authorized users.

## Introduction

A common approach to evaluate the impacts of natural or human-induced perturbations on ecosystems where the allocation of treatment and control sites cannot be assigned randomly is a before-after-control-impact/treatment (BACI) design (Eberhardt 1976; Green 1979). A variety of BACI designs have been proposed to draw inferences about impacts (e.g., BACIPS, MBACI, and beyond-BACI, following the nomenclature of Downes et al. 2002). A primary example and impetus for development of the method was evaluation of the impacts of a nuclear power plant on many ecological response variables, from zooplankton abundance (Mathur et al. 1980; Bence et al. 1996) to communities of macroinvertebrates and related physical variables (Schroeter et al. 1993). BACI designs continue to be used to evaluate impacts from natural perturbations (Russell et al. 2015) and management actions (Desrosiers et al. 2006; Louhi et al. 2010; Hanisch et al. 2013), as well as for a wide variety of smaller-scale field experiments, including evaluating restoration actions (Rumbold et al. 2001; Muotka and Syrjänen 2007; Bousquin and Colee 2014). Similar to studies of larger-scale impacts, efficiently evaluating restoration activities is complicated because, in many cases, restoration actions cannot be implemented in randomly selected locations owing to factors such as access requirements and land ownership, and replication is often restricted due to limited numbers of potential restoration sites, cost of restoration, and logistical constraints.

Analysis of BACI designs has conventionally involved the use of general linear models (e.g., analysis of variance, see Downes et al. 2002) or the use of intervention analyses (Carpenter et al. 1989; Stewart-Oaten and Bence 2001). A particularly useful modification is where impacted and control sites are treated as fixed effects and sampling is conducted at simultaneous (paired) time periods in treatment and control sites before and after perturbation (BACIPS; Stewart-Oaten et al. 1986; Underwood 1994). Treatment effects for BACIPS designs are often estimated as the mean difference between treatment and control sites after the treatment minus the mean difference between treatment and control sites before the treatment $\left({\overline{d}}_{\mathrm{treat}\hbox{-} \mathrm{control}\ \mathrm{after}}-{\overline{d}}_{\mathrm{treat}\hbox{-} \mathrm{control}\ \mathrm{before}}\right)$ (Stewart-Oaten et al. 1986; Bence et al. 1996), or via a treatment (control–treatment) × time (before–after) interaction term (Russell et al. 2009; Popescu et al. 2012). This design allows treatment impacts to be distinguished from background time effects shared by all sites, as well as from background differences between treatment and control sites (Popescu et al. 2012). In essence, this design controls for spatial differences between treatment and control sites such that they do not have to be identical. Because of its applicability to restoration field experiments, here we focus on the BACIPS design.

Although the design works well for testing for perturbation effects in field experiments, the results (i.e., treatment minus control difference or significant interaction term) from BACIPS designs analyzed using frequentist statistical approaches typically lack meaningful probabilistic interpretation and are thus not easily understood by nonscientific audiences (Eberhardt 1976; Crome et al. 1996). There is a continuum of interpretability of frequentist results, with *P* values being perhaps the least understandable to a lay audience and effect sizes and their confidence intervals being more accessible. However, the interpretation of confidence interval is also not intuitive: the interval the unknown true mean change would fall between at the frequency of the confidence level if the experiment were repeated. Bayesian approaches have advantages for interpretation. Because the Bayesian approach is explicitly conditioned on the observed data, Bayesian inference provides direct probability assessments of the response parameter that are more straightforward to interpret (e.g., probability of a % increase or decrease in population size) (Crome et al. 1996; Wade 2000). Moreover, the Bayesian approach has the flexibility to use posterior distributions to estimate a variety of comparisons (Wade 2000) and to report the probability of observing a range of effects sizes (Gelman et al. 2004; Kery 2010; King et al. 2010). In addition, by conditioning on the data, not a specific hypothesis, and providing inference about a range of effect sizes, a Bayesian approach reduces the potential for type I and type II errors, a long-standing criticism of the analysis of BACI data (Mapstone 1995; Murtaugh 2002). If study results, particularly contentious ones, can be conveyed in a manner that is accessible, yet accurate, to both scientific and lay audiences alike, they are far more likely to be embraced by resource managers (Crome et al. 1996).

Here, we present a method with Bayesian interpretability to evaluate responses of treatment sites to natural perturbations or management actions via an adaptable proportional response variable combined with a Bayesian hierarchical model and Markov chain Monte Carlo (MCMC) sampling to estimate the probability of observing different effect sizes. To demonstrate these techniques and highlight their usefulness for evaluating restoration actions, we use a dataset from a BACIPS field experiment combined with a Bayesian MCMC approach to evaluate the effectiveness of a river restoration project to increase juvenile steelhead (*Oncorhynchus mykiss*) survival and density. While we use a particular study design here (BACIPS), this approach can be readily adapted for a wide variety of statistical study designs, from simple (e.g., single factor ANOVA, paired *t* test) to more complicated block designs (e.g., crossover, split-plot).

## Materials and methods

### Study area and field sampling

**Fig. 1:**
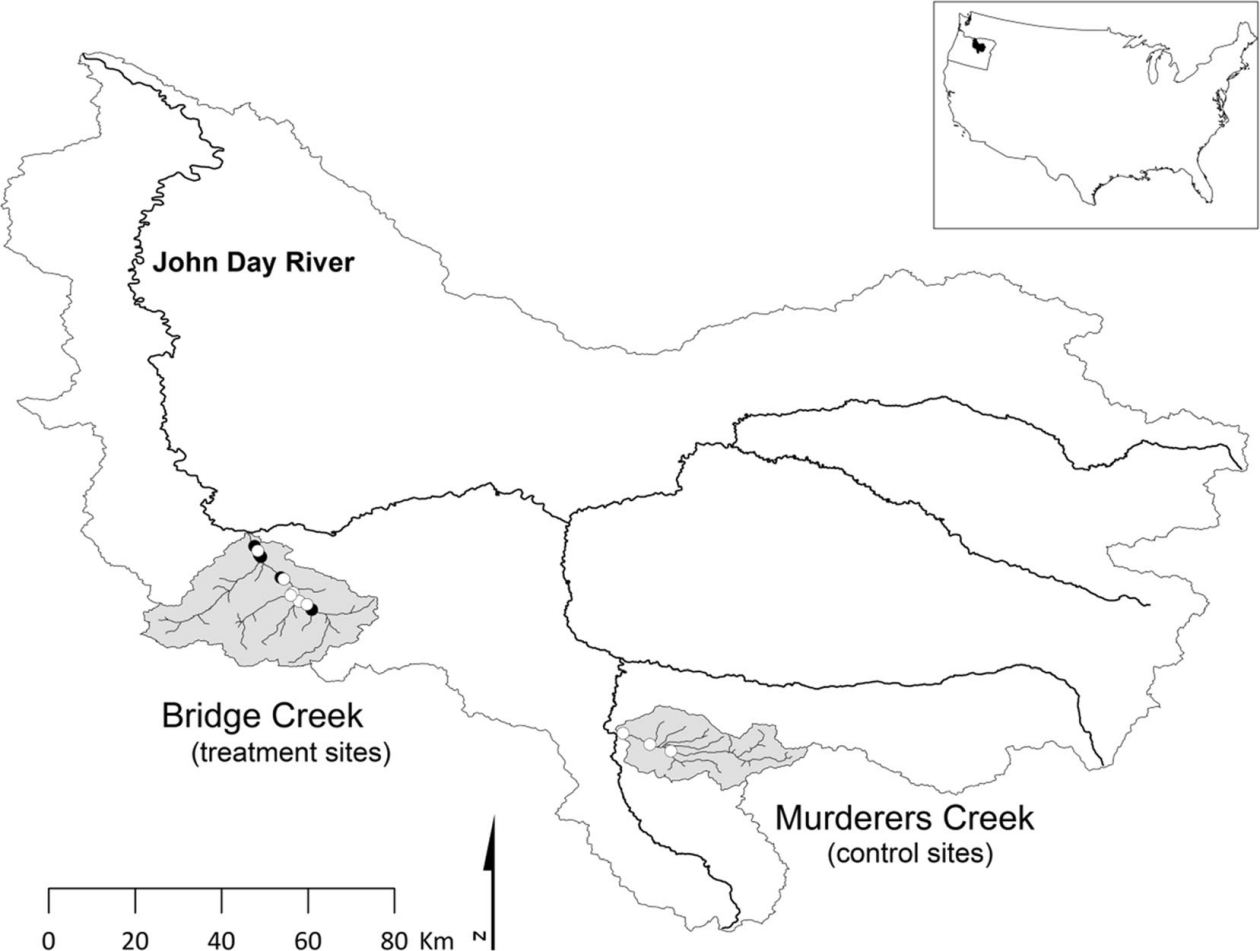
Study sites in Bridge and Murderers creeks, located in the John Day River Basin, OR, USA. *Open circles* indicate sites used for both analysis of abundance and survival data, while *filled circles* indicate additional sites used only for analysis of survival data

The data used to demonstrate this analysis method were collected in Bridge Creek and Murderers Creek, tributaries to the John Day River and part of the larger Columbia River Basin (Fig. 1). The John Day River is occupied by federally threatened steelhead that spawn in both Bridge and Murderers creeks. After emergence, these tributaries provide rearing habitat for juvenile steelhead (anadromous life history of *O. mykiss*) as well as rainbow trout (resident life history of *O. mykiss*). Owing to historical land use practices, extensive portions of Bridge Creek have undergone substantial down-cutting, resulting in a narrow incised straightened channel that lacks habitat complexity necessary to support robust *O. mykiss* populations. In an attempt to aggrade the channel by capturing fine sediments and ultimately increase channel complexity, beaver dam analogs (BDAs) spanning tributary channels were constructed within four treatment reaches on Bridge Creek (Pollock et al. 2014; Bouwes et al. 2016). This restoration strategy assumed that BDAs and subsequent colonization by resident beaver would increase both the total surface area available to juvenile *O. mykiss* as well as increase habitat complexity available for juvenile fish (Bouwes et al. 2016). The BDAs were installed during December 2009. The control watershed, Murderers Creek, was chosen because it is a stream of similar size, discharge, and gradient and resides in the same biome as the treatment watershed (Bridge Creek).

Mark-reencounter sampling was conducted from January 2007 through September 2012 to estimate seasonal survival and density of juvenile steelhead. More complete descriptions of the study area and sampling methods are provided in Tattam et al. (2013) and Pollock et al. (2012). Juvenile steelhead were captured by electroshocking at permanent sites that ranged from 500 to 1000 m long. All steelhead greater than 60 mm were tagged with passive integrated transponders (PITs) and released at the site of capture. In the Bridge Creek, the treatment watershed, 13 sites (four in the treatment reaches and nine in nontreated reaches) were sampled throughout watershed over the study period. Because fish can move between sites, we considered all sites in the watershed to be treatment sites. In the lower portion of Bridge Creek, in four of the sites, an insufficient number of fish could be tagged to obtain accurate density estimates, and thus were not included in the density analyses. In Murderers Creek, which served as the control watershed, three sites were sampled in its lower portion (Fig. 1). Sites were sampled on two consecutive days (closed-capture sessions), and each site was revisited during three seasons, generally representing summer (June), fall (September), and winter (December–January). The entire sampling period within a season was relatively short, averaging 1 to 2 weeks (in order to sample all sites), with each site having approximately the same period of time between closed-capture sessions (although period length varied from season to season). This yielded three biologically relevant seasons for survival rates—summer (June–September), fall (October–December), and winter/spring (January–May; Table 1)—and three estimates of population abundance for each year (Table 1).

**Table 1:** Seasonal periods over which juvenile steelhead survival was estimated on Bridge and Murderers creeks before and after construction of BDAs on Bridge Creek, OR

Year	Season	Impact period	Start	End	Total days
2007	Spring	Before	June 4, 2007	September 1, 2007	89
2007	Fall	Before	September 1, 2007	November 28, 2007	88
2008	Winter	Before	November 28, 2007	June 1, 2008	186
2008	Spring	Before	June 1, 2008	September 1, 2008	92
2008	Fall	Before	September 1, 2008	December 12, 2008	102
2009	Winter	Before	December 12, 2008	June 10, 2009	180
2009	Spring	Before	June 10, 2009	September 14, 2009	96
2009	Fall	Before	September 14, 2009	January 22, 2010	130
2010	Winter	After	January 22, 2010	June 9, 2010	138
2010	Spring	After	June 9, 2010	September 13, 2010	96
2010	Fall	After	September 13, 2010	January 24, 2011	133
2011	Winter	After	January 24, 2011	June 24, 2011	151
2011	Spring	After	June 24, 2011	September 16, 2011	84
2011	Fall	After	September 16, 2011	January 10, 2012	116
2012	Winter	After	January 10, 2012	June 21, 2012	163
2012	Spring	After	June 21, 2012	September 11, 2012	82

### Statistical methods

The overall goal of this study is to demonstrate the use of a Bayesian approach to estimate the probability of observing different restoration treatment effect sizes for a BACIPS study for parameters of different scales (i.e., one constrained 0–1 and the other not). To do this, we generated the best estimates of juvenile steelhead survival and density before and after the restoration action was implemented on treatment and control watersheds, and then used these to estimate probabilities of increases or decreases in response to the restoration.

### Survival

We generated encounter histories for each individual PIT-tagged fish from active tagging, mobile antenna surveys, and continuous detections from passive instream antenna (PIA) arrays, located in four locations in Bridge Creek and one location in Murderers Creek. Separate encounter histories were generated for treatment and control watersheds. Because continuously collected detections by PIAs were an important method for reencountering PIT-tagged fish, we used the Barker model (Barker 1997) rather than a Cormack-Jolly-Seber (CJS) model to estimate survival. We censored encounter histories for fish detected leaving tributaries (resighted at terminal antenna arrays) to reduce bias in survival estimates owing to permanent emigration (Horton and Letcher 2008; Conner et al. 2014). We used Program MARK (White and Burnham 1999; White et al. 2001) to analyze these data.

Because seasonal periods (*t*) were of slightly unequal length (Table 1), we standardized survival estimates ($\widehat{S}$_*t*_) to a 3-month period (e.g., $\widehat{S}$_*t*_ = 0.6 is probability animal survived for 3 months) using unequal time intervals in Program MARK (White et al. 2001). There were eight seasons pre-restoration and seven seasons post-restoration over which survival was estimated (the last survival estimate was not used because it was confounded with resight probabilities). Because the study was designed as a BACIPS study, we analyzed data from treatment and control watersheds separately and left estimates of S_*t*_ unconstrained in all models. That is, *S*_*t*_ was estimated for each season before and after implementation of the BDAs for control ($\widehat{S}$_*t* control_) and treatment watersheds ($\widehat{S}$_*t* treat_). Before proceeding with a hierarchical model for *S*_*t*_ using a Bayesian approach, we wanted to find the best model for the other parameters in the Barker model (e.g., p, R, F, etc.). To this end, we constructed a series of more parsimonious models for all other model parameters in the Barker model (see Supplemental Information for the details of model construction and model sets) and used the top model structure (i.e., model with the lowest AICc; Lebreton et al. 1992; Burnham and Anderson 2002) from which to estimate posterior distributions of S_*t*_. Note that we did not use model averaging as part of the analysis because it would be a much more complex approach. That is, we would need to do MCMC simulations for each model in the set, and then apply the model weight and average across the 5000 simulations for each model, and then do the averaging across time periods (and sampling sites for abundance); this was beyond the scope of what we wanted to highlight for this paper.

We used a Bayesian hierarchical model with hyperdistributions to estimate mean survival and get “shrinkage” estimates for *S*_*t*_ for treatment and control groups by before and after periods $\left(\mathrm{e}.\mathrm{g}.,{\tilde{S}}_{\mathrm{control},\mathrm{before},}{\tilde{S}}_{\mathrm{treat},\mathrm{after}}\right)$. That is, we specified four hyperparameters. For these hyperdistributions, we used MCMC sampling implemented in Program MARK to generate posterior distributions of $\widehat{S}$_*t* control_ and $\widehat{S}$_*t* treat_, which were shrinkage estimates that we used for estimating ratios to evaluate the treatment effect as described below. Because this was the first time, we analyzed the data using a BACI model and because we used different subsets of the data for previous analyses, we used uninformative “flat” priors for the hyperpriors of the four estimates of mean survival ($\overset{\sim }{S}$):

$$ \tilde{S}\sim N\left(\mu, \sigma \right) $$

$$ \mu \sim N\left(0,100\right) $$

$$ {}^1{/}_{\upsigma^2}\sim \gamma \left(0.001,0.001\right) $$

where *γ* represents a gamma distribution. In addition to the parameters included in hyperdistributions, there were additional “nuisance” parameters (*θ*) in the Barker model (e.g., recapture probability, resighting probability, etc; see Supplemental information for Barker model parameter specification). These parameters also require a prior distribution. All additional model parameters were logit transformed to constrain the real estimates to be between 0 and 1. For these, we used a normal prior on the logit scale:

$$ logit\kern0.28em \left(\theta \right)\sim N\left(0,1.75\right), $$

which is a relatively flat prior when back transformed to the real 0–1 scale (2.5th and 97.5th percentiles of approximately 0.02 and 0.98, with a uniform distribution between those percentiles when back transformed). We assessed convergence of the Markov chains by visual inspection of the trace of MCMC chains of the posterior samples of the parameters and by using the Gelman-Rubin statistic, R-hat (Gelman et al. 2004). For each parameter, we used ten chains of 1000 each and used a threshold of R-hat <1.1 to indicate adequate sampling of the posterior distribution. Based on diagnostics in Program MARK’s MCMC routine (Cooch and White 2016), we determined posterior distributions needed to be thinned and accordingly saved every sixth sample to achieve first-order Markovian independence. We used 1000 burn in samples and kept 5000 samples after thinning.

To estimate treatment effects for this BACIPS study, we used the posterior distributions of $\widehat{S}$_*t*_ to estimate the posterior distribution of the ratio of treatment to control watersheds (${\widehat{R}}_{t\ t\Big|c}$) as ${\widehat{R}}_{t\ t\Big|c}$= $\widehat{S}$_*t* treat_/ $\widehat{S}$_*t* control_) for each time period. We then estimated the posterior distribution of the treatment effect for survival (${\widehat{R}}_{S\ \mathrm{BACI}}$) as ${\widehat{R}}_{S\ \mathrm{BACI}}$ = ${\overset{-}{\widehat{R}}}_{t\Big|c\ \mathrm{after}}$/${\overset{-}{\widehat{R}}}_{t\Big|c\ \mathrm{before}}$. That is, for each MCMC sample, we calculated the mean ratio from the seven seasons after the BDAs were installed and the mean ratio from the eight seasons before the BDAs were installed, and then divided them. Note that because the ratios were log-normally distributed, we did all calculations on the log scale, and then back transformed the final ${\widehat{R}}_{S\ \mathrm{BACI}}$ for each MCMC sample. We estimated the median and 2.5 and 97.5 percentiles for the distribution of ${\widehat{R}}_{S\ \mathrm{BACI}}$.

### Density

Abundance for each site was estimated from the two closed-capture sessions, which occurred at the start of each of the seasonal time periods described above for survival (Table 1), except for one additional capture session that occurred pre-treatment in January (winter) 2007. Thus, for each closed-capture session, a fish could have a 10 (captured the first session but not captured the second session), 11 (captured the first session and captured the second session), or 01 (not captured the first session but captured the second session) encounter history. We summarized these encounter histories for each of the two closed-capture sessions across sites for each time period (season). There were 18 abundance estimates, 10 before and 8 after BDAs were installed for each site. There were three additional abundance estimates relative to survival because there was an additional closed-capture session at the start of the study, survival could not be estimated for the last seasonal period, as discussed above, and survival is an interval estimate (i.e., there is one survival estimate between two closed-capture sessions/estimates).

We used a Bayesian MCMC approach to generate a posterior distribution of abundance (*N*) for each site and time period based on number of unique individuals captured (*n*) and capture probability. We used closed-capture model *M*_0_ (Otis et al. 1978) and a data augmentation procedure (Royle and Dorazio 2012) for closed-capture models following Royle et al. (2007). We augmented each sample (*n*) by 500 (*z*) because this was more than twice any empirical abundance estimate for any of the study sites. This augmentation provided, in essence, a relatively uninformative prior (i.e., *M* = *z* + *n*, and *N* ∼ DU(0, *M*) where DU = discrete uniform distribution; for details, see Royle and Dorazio 2012). To obtain a posterior distribution of site abundances, we used WinBUGS (Lunn et al. 2000), called from matbugs (available from http://code.google.com/p/matbugs/) in MATLAB (v. R2012b; MATLAB___8.0 2012). We ran model *M*_0_ for each site and time period using 20,000 MCMC samples after discarding the first 1000 samples as burn in for each of three chains. We thinned by saving every third sample to reduce autocorrelations between samples; thus, we retained 5000 samples. We determined if the Markov chains converged using the Gelman-Rubin statistic (called Brooks-Gelman-Rubin statistics in WinBUGS), R-hat (Gelman et al. 2004). For each site and period, we used three chains of 5000 each and used a threshold of R-hat <1.1 for *N* to indicate adequate sampling of the posterior distribution.

From the posterior distributions of abundance, we generated posterior distributions of density (*D*) for each site as fish/100 m by dividing each abundance estimate by the site length and then standardizing to 100 m. To generate one estimate per time period for treatment and control sites, we averaged the log of the density estimates across treatment and control sites for each time period. We did this for each MCMC sample to generate a posterior distribution of average density for treatment and control watersheds for each period. Then, similar to survival, we used the ratio of these estimates to estimate treatment effects for this BACIPS study. That is, we calculated the ratio of the treatment to control watersheds (${\widehat{R}}_{t\ t\Big|c}$) as ${\widehat{R}}_{t\ t\Big|c}$ = $\widehat{\overset{-}{D}}$_*t* treat_/ $\widehat{\overset{-}{D}}$_*t* control_) for each time period. We then estimated the posterior distribution of the treatment effect for density (${\widehat{R}}_{D\ \mathrm{BACI}}$) as ${\widehat{R}}_{D\ \mathrm{BACI}}$ = ${\overset{-}{\widehat{R}}}_{t\Big|c\ \mathrm{after}}$/${\overset{-}{\widehat{R}}}_{t\Big|c\ \mathrm{before}}$. That is, for each MCMC sample, we calculated the mean ratio from the eight seasonal periods after BDAs were installed and the mean ratio from the ten seasonal periods before the BDAs were installed, and then divided them. Note that because the ratios were log-normally distributed, we did all calculations on the log scale, and then back transformed the final ${\widehat{R}}_{D\ \mathrm{BACI}}$ for each MCMC sample. We estimated the median and 2.5 and 97.5 percentiles for the distribution of ${\widehat{R}}_{D\ \mathrm{BACI}}$.

## Results

**Fig. 2:**
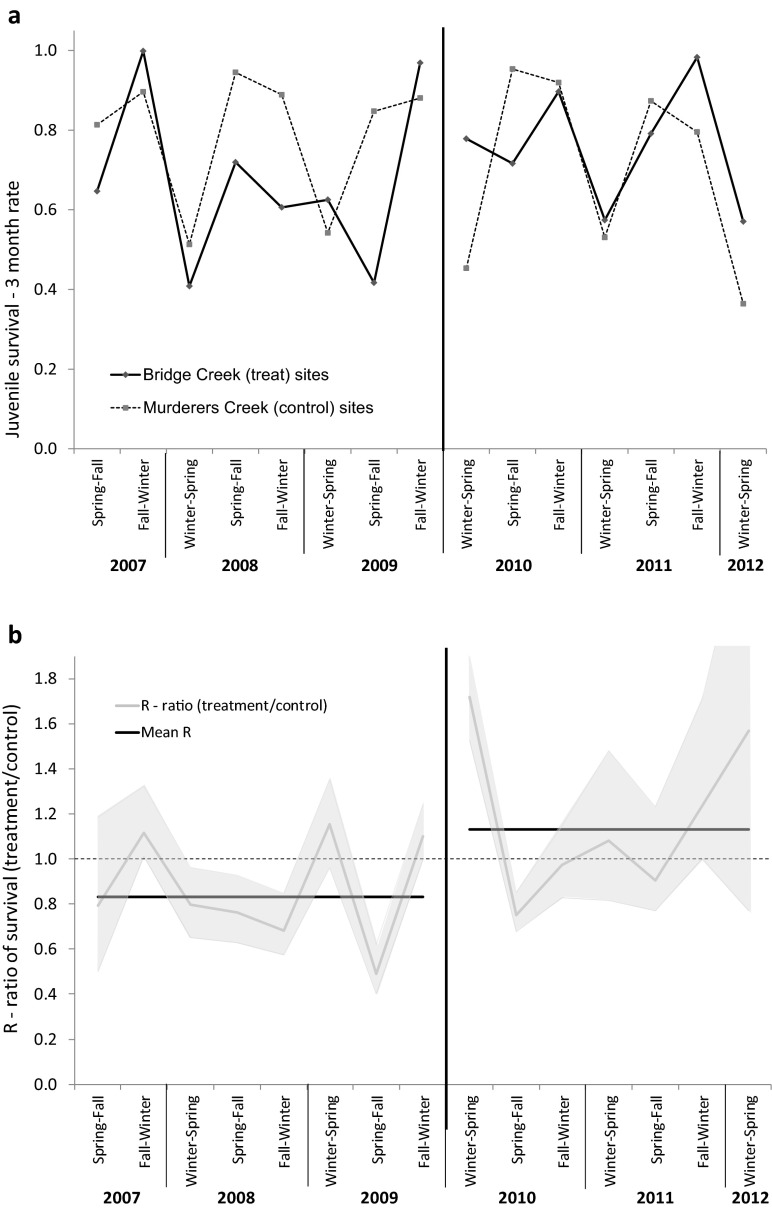
The **a** 3-month juvenile survival probability of steelhead and **b** the ratio of survival on treatment sites to control sites for each period (*gray line*), and the geometric mean of the ratios (*black line*) before and after BDAs were installed in Bridge Creek, OR, 2007–2012. For **b**, the *shaded area* represents the 95 % credible interval and the *dashed line* represents no treatment effect between control and treatment sites during the before or after periods; *values above the dashed line* indicate survival was higher on treatment sites relative to control sites, and *values below* indicate survival was lower on treatment sites relative to control sites

We used 5728 and 2410 marked juvenile steelhead on treatment and control watersheds before beaver dam analogs were installed, and 7892 and 2227 after, for the analysis of survival. The Barker global model of survival fit adequately (i.e., there was not significant overdispersion or underdispersion); $\widehat{c}$ = 1.15 for the control watershed data set and $\widehat{c}$ = 1.21 for the treatment watershed. Because $\widehat{c}$ > 1, we corrected and used QAICc for subsequent survival analyses. Survival estimates from the top-ranked model showed seasonal temporal variation for treatment and control watersheds, with the control watershed showing a consistent pattern of lower winter and higher spring and fall survival (Fig. 2a). Despite the temporal variation, the average survival on the treatment watershed increased after installation of the BDAs, relative to the control watershed (Fig. 2b); ${\overset{-}{\widehat{R}}}_{t\Big|c\ \mathrm{before}}$ = 0.83 and ${\overset{-}{\widehat{R}}}_{t\Big|c\ \mathrm{after}}$ = 1.13, which resulted in an overall treatment effect ${\widehat{R}}_{S\ \mathrm{BACI}}$ = 1.36. This indicates that survival on the treatment watershed increased, on average, 36 % after the beaver dam analogs were installed, relative to survival on the control watershed.

**Fig. 3:**
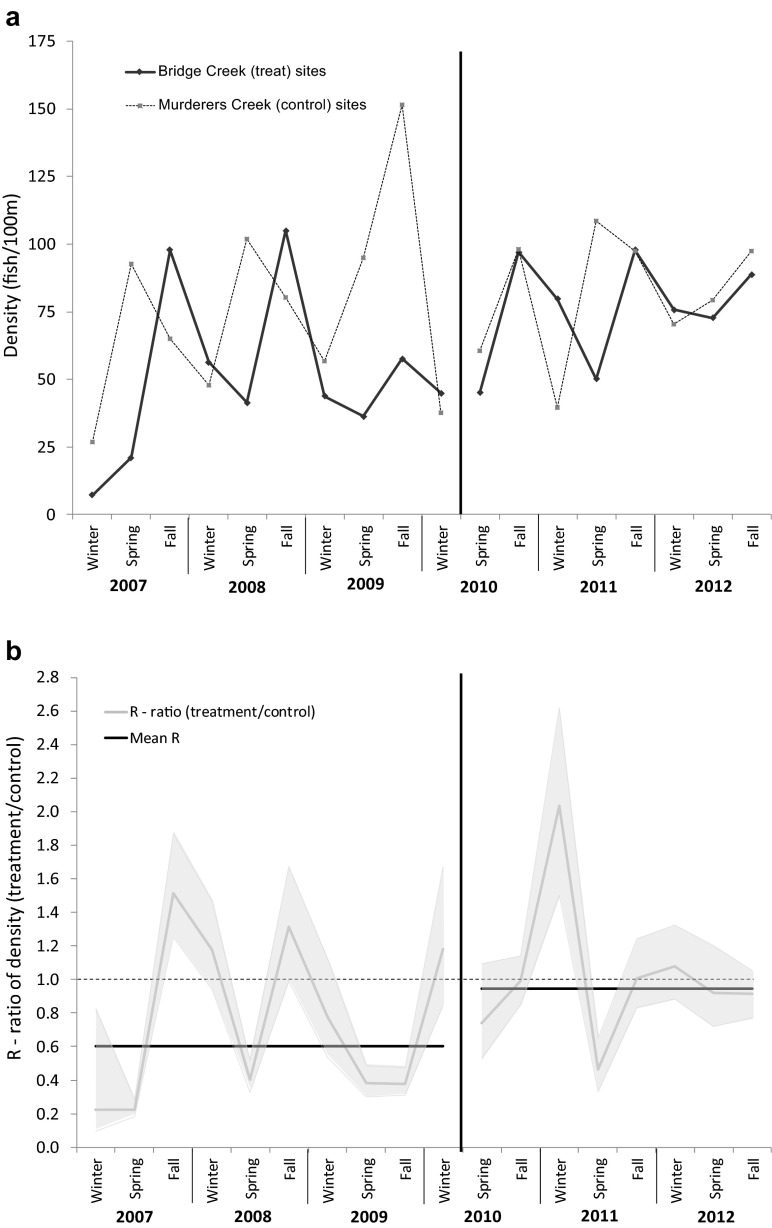
The **a** density of steelhead and **b** the ratio of density on treatment sites to the control sites for each period (*gray line*), and the geometric mean of the ratios (*black line*) before and after BDAs were installed in Bridge Creek, OR, 2007–2012. For **b**, the shaded area represents the 95 % credible interval and the *dashed line* represents no treatment effect during the before or after periods; *values above the dashed line* indicate density was higher on treatment sites relative to control sites, and *values below* indicate density was lower on treatment sites relative to control sites

We used 4441 and 2440 marked juvenile steelhead on treatment and control sites before BDAs were installed, and 4955 and 1636 after for the analysis of density. Recapture rates were very similar for treatment and control sites both before (0.12 for both treatment and control) and after the installation of BDAs (0.07 treatment and 0.10 control). Similar to survival, density estimates showed seasonal variation for treatment and control watersheds, with the control watershed showing a consistent pattern of lower winter and higher spring and fall density (Fig. 3a). The average density on the treatment watershed also increased after installation of the BDAs, relative to the control watershed (Fig. 3b); ${\overset{-}{\widehat{R}}}_{t\Big|c\ \mathrm{before}}$ = 0.60 and ${\overset{-}{\widehat{R}}}_{t\Big|c\ \mathrm{after}}$ = 0.95, which resulted in an overall treatment effect ${\widehat{R}}_{D\ \mathrm{BACI}}$ = 1.58. This indicates that density on treatment watershed increased, on average, 58 % after the BDAs were installed, relative to density on the control watershed.

**Fig. 4:**
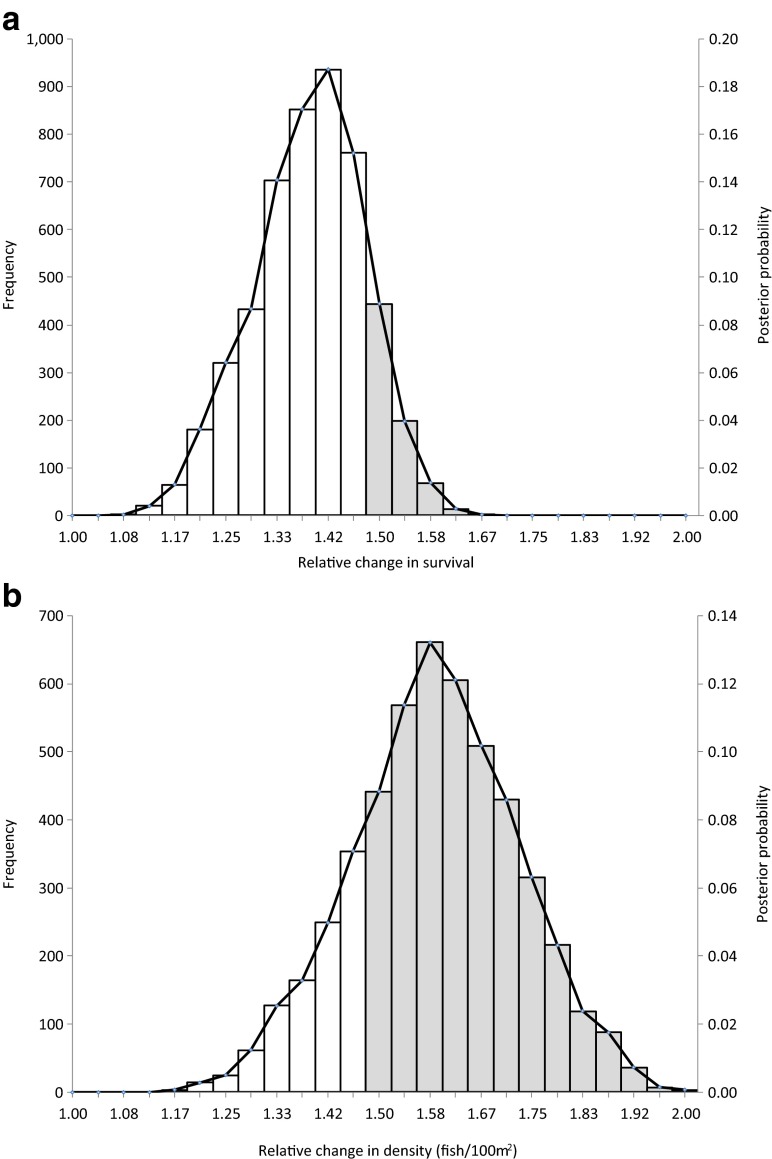
Distribution of the relative change (${\widehat{R}}_{\mathrm{BACI}}$) in **a** juvenile survival and **b** density of steelhead in Bridge Creek, OR, 2007–2012. The relative change is the ratio of the geometric mean of the ratios (for each period) of survival and density on treatment sites (Bridge Creek) relative to control sites (Murderers Creek), with the ratio after divided by the ratio before BDAs were installed. Relative change >1 indicates an increase on treatment sites relative to control sites after the BDAs were installed relative to before the dams were installed, while values of <1 indicate a decrease on treatment sites relative to control sites after the BDAs were installed relative to before they were installed. The *shaded columns* are an example showing the probability that survival or density increased by ≥50 %

The posterior distributions of ${\widehat{R}}_{S\ \mathrm{BACI}}$ and ${\widehat{R}}_{D\ \mathrm{BACI}}$ indicate a zero probability that survival or density decreased after the BDAs were installed (Fig. 4). Note that a decrease would have been indicated by ${\widehat{R}}_{\mathrm{BACI}}$ <1. After BDAs were installed, the probability of an increase of ≥30 % on the treatment watershed relative to the control watershed was high for both survival (0.88) and abundance (0.99; Table 2). The largest difference in the impact of the BDAs was for the probability of a ≥50 % increase; for survival it was only 0.17, while for abundance it was 0.82 (Table 2 and shown by shaded areas, Fig. 4). The posterior distribution of ${\widehat{R}}_{D\ \mathrm{BACI}}$ was shifted to the right relative to ${\widehat{R}}_{S\ \mathrm{BACI}}$, and so density showed higher probabilities of greater potential increases after the installation of BDAs compared to survival (Fig. 4 and Table 2). The variation in relative change was also greater for density than survival; the posterior distribution CI width was 43 % wider for density compared to survival (Fig. 4).

**Table 2:** Estimates of the probability juvenile survival and density increased or decreased a given percentage after BDAs were installed on study sites in Bridge Creek, OR, 2007–2012

Parameter	≥0 %	≥20 %	≥30 %	≥50 %	≥100 %
Increase					
Survival	1.00^a^	0.99	0.88	0.17	0.00
Density	1.00	1.00	0.99	0.82	0.00
Decrease					
Survival	0.00	0.00	0.00	0.00	0.00
Density	0.00	0.00	0.00	0.00	0.00

^a^Probabilities are based on a posterior distribution of relative change (${\widehat{R}}_{\mathrm{BACI}}$), which is the geometric mean of the ratios (for each period) of survival and density on the treatment watershed (Bridge Creek) relative to the control watershed (Murderers Creek), with the ratio after divided by the ratio before BDAs were installed

## Discussion

Our results demonstrate a useful extension of Bayesian methods to estimate probabilities of different effect sizes for BACI style study designs. Here, for two different population parameters that had output that differed in distribution and magnitude, we quantified the probability that restoration had a negative or positive impact. In addition, we can readily evaluate different levels of impact. For example, the probability that BDAs increased both survival and density of juvenile steelhead by ≥50 % was 0.17 and 0.82, respectively (Table 2), and we can compare this to the probability of a more moderate increase of ≥30 % (0.88 and 0.99, respectively; Table 2). Indeed, the output metrics from a posterior distribution are flexible, and metrics such as presented here are intuitive to restoration and other management concerns and well adapted for decision making (Wade 2000).

The combination of a ratio test statistic and the Bayesian approach yields results that are directly applicable to restoration and management questions (as well as for evaluating natural perturbation impacts). First, using a Bayesian MCMC approach to estimate this test statistic is particularly useful because the posterior probability distribution of the treatment effect (${\widehat{R}}_{\mathrm{BACI}}$) can be used to directly draw inferences about the probability that there was a change in the response variable, given the observed data (Crome et al. 1996). Secondly, using a Bayesian approach provides accurate estimates of variation for the ratios (or any contrast, including nonlinear contrasts, of interest), whereas approaches to estimate variance from combined or transformed variables, such as the Delta method, can yield poor estimates where the function is nonlinear (Cooch and White 2016) or when the variance in the measured response is relatively large (e.g., *CV* > 20–50 %; Zhou 2002).

Additionally, using a test statistic that is a ratio of treatment to control observations provides directly interpretable effects in terms of the percent response of treatment sites, relative to control sites, after a restoration action was implemented relative to before period (${\widehat{R}}_{\mathrm{BACI}}$). Thus, if ${\widehat{R}}_{\mathrm{BACI}}$ = 1.28, there was a 28 % increase in the response variable in the treatment watershed after manipulation. As ratios provide an interpretation based on proportional responses, effect sizes are directly comparable across multiple response variables, relative to management targets or biologically reasonable responses, which can vary in both magnitude (daily growth versus animal abundance) and domain (e.g., survival [0–1] versus density [positive numbers]). Thus, while the mechanisms for changes in density (reproduction, mortality, immigration, emigration) and survival (mortality) following manipulation differ significantly, a ratio test statistic can be used to draw inference about the probability a manipulation would achieve management goals with different effect sizes across response variables in a consistent and easily comparable manner. For example, here we can easily compare the probabilities of restoration targets such as a 50 % increase in density (0.82) and a 20 % increase in survival (0.99). However, while a ratio test statistic provides a useful metric to quantify changes in a response variable following manipulations and facilitates comparison of observed effect sizes from multiple response variables or potential study targets, it does not directly imply biological significance of that response to a population of interest. For example, the impacts of a 20 % increase in juvenile steelhead survival following BDA installation on the population as a whole is dependent on the survival rate prior to manipulation and would need to be evaluated using a population projection model, to put it within the context of other demographic constraints.

Since the initial proposal of before-after (Box and Tiao 1965) and BACI (Green 1979) designs, the development of more sophisticated study designs, including the paired BACIPS (Stewart-Oaten et al. 1986), beyond-BACI (Underwood 1994), and multiple BACI (MBACI; Keough and Quinn 2000), has spawned an unresolved debate about the most appropriate study design to draw inferences from field studies involving nonrandom assignment of unreplicated treatments (Reckhow 1990; Underwood and Chapman 2003; Webb et al. 2010). However, studies are often constrained by resources, the existence of suitable reference sites, and the ability to collect data at reference and impact sites both before and after a perturbation occurs for a long enough time series to have power to detect a change at impact sites. These constraints can result in high rates of rejecting the null hypothesis when in fact there was no impact (type I error; Murtaugh 2002), or sometimes accepting a null hypothesis when in fact there was an impact (type II error; Benedetti-Cecchi 2001). While it does not mitigate the importance of good study design, the Bayesian approach we describe partially alleviates the concern over type I and II errors by directly estimating the probability of observing an effect size (or range of effect sizes), conditional on the observed data, as opposed to probability of observing the data (or more extreme data), conditional on a specific hypothesis and assumptions that may not be satisfied by the study design and data.

We concur with recent assertions that estimation of effect size is more important, and more informative, than significance testing for management applications (Stewart-Oaten et al. 1992; Mapstone 1995; Crome et al. 1996). Manipulation of Bayesian posterior distributions allows analysts to determine the probability of observing any effect size of interest, or contrast the probability of effect sizes that differ in magnitude. For example, Bayesian approaches have been used to determine the probability that mean pH in Adirondack lakes increased by ≥10 % during a 7-year study period (Reckhow 1990), California spotted owl populations increased or decreased by ≥0, 30, and 50 % during a 20-year study period (Conner et al. 2013), bird community composition changed by greater than or equal to −25, 0, and 25 % owing to logging practices (Crome et al. 1996), and that there was a ≥75 % reduction in occupancy across sites after a hurricane (Russell et al. 2015). Thus, analyses can be readily framed to report the probability that a change of a magnitude deemed to be important to managers/policymakers has occurred. In contrast, the question asked by a classical hypothesis test is whether the test statistic calculated from the sample mean was unusual in comparison to what we would expect to calculate if there was no change. Inferences drawn about significant effect sizes from hypothesis-driven approaches can be subject to questions of biological significance and are often difficult to interpret with regard to management goals or conservation targets.

The combination of a ratio test statistic and Bayesian approach can easily be generalized to wide variety of study designs and provide an answer to the main study question—“How much impact (positive or negative) did the restoration action (or natural perturbation) have?” While determining restoration management effects in a field setting is the main focus of this paper, the ratio and Bayesian approach could be applied to controlled experiments or treatment contrasts of other response variables as well. Primary to adapting this approach to other applications is defining the set of models that capture the study design and processes determining the response variables; in addition, this approach has the additional advantage that priors can be incorporated, if the data are available, for Bayesian analysis (see Wade 2000; Hobbs and Hooten 2015). Indeed, manipulation of posterior distributions can facilitate inferences drawn using a ratio test statistic. For example, Kimball et al. (2014) describe a split-plot designed experiment to evaluate the impacts of water and nitrogen input on percent cover of native shrubs. They provide estimates of percent native cover for different input levels, but could recast the results to describe the probability that water reduction (emulating drought conditions) decreased the percent native cover by 50 %, or some relevant ecological or management threshold. For other non-BACI study designs in less controlled field experiments, Bayesian methods have been used to describe widely ranging response variables of interest, including growth of individuals (Tanentzap et al. 2014; Tang et al. 2014), occupancy of species across a landscape (Russell et al. 2009), structure of physical habitat (Wallis et al. 2008), and biochemical makeup of terrestrial and aquatic systems (Qian et al. 2005; Larssen et al. 2006; Tanentzap et al. 2014). Such models can easily be adapted to provide posterior distributions that facilitate ratio contrasts between treatment and control experimental units for either controlled experiments, as well as field studies based on non-BACI study designs.

### Management applications

BACI designs have been used to evaluate a variety of field experiments where randomization of treatment and/or control sites is not possible (Skilleter et al. 2006; Conner et al. 2007; Pitcher et al. 2009; Russell et al. 2015). While any management action can be evaluated with this approach, we believe it has particular relevance to restoration activities. Ecologists and managers tasked with conserving wildlife species, especially those showing declining population trends, often employ habitat restoration to enhance population vital rates and increase abundance. However, the majority of restoration activities go unevaluated (Bernhardt et al. 2005), while those that have been evaluated show varying degrees of success (Thompson 2006; Roni et al. 2008; Stewart et al. 2009; Whiteway et al. 2010). As a result, information is sparse as to which restoration activities recover declining populations as well as the extent to which restoration actions affect population responses. The BACI design can yield inference about impacts of restoration across broad scales (Underwood 1994; Keough and Mapstone 1995; Stewart-Oaten and Bence 2001), but to date has yet to be incorporated in many evaluations of restoration effectiveness (Miao et al. 2009). This is particularly unfortunate because, in many cases, the planning and permitting process involved with restoration activities provide an opportunity to initiate carefully designed BACI type studies, providing a time series of data both before and after restoration activities occur. In addition, the analysis of BACI data using a Bayesian approach is particularly well suited for the evaluation of restoration effectiveness as the inferences drawn about restoration impacts can be easily understood by many stakeholders.

The combination of a ratio test statistic and Bayesian approach we outline here, in conjunction with carefully a designed BACIPS study, provides ecologists and managers with an elegant means to quantify the probability of various effect sizes of interest, which can be a useful for managers trying to balance trade-offs between costly management actions and conservation of wildlife populations. Moreover, this approach provides results that are easily understandable to ecologists, managers, and stakeholders with a nonscientific background alike. We hope this approach will be useful for field ecologists and managers involved in restoration studies, but will also have wider application for any field study that suffers from a lack of adequate randomization and replication.

## Electronic supplementary material

ESM 1 (DOCX 33 kb)

ESM 2 (M 7 kb)

ESM 3 (SAS 4 kb)
