# Supplementary material for: Evaluating impacts using a BACI design, ratios, and a Bayesian approach with a focus on restoration
Source: Environ Monit Assess. 2016 Sep 8;188(10):555. doi: 10.1007/s10661-016-5526-6 (PMC5016564; doi:10.1007/s10661-016-5526-6)
Supplement: Supplementary file 1 — (DOCX 33 kb) [file 10661_2016_5526_MOESM1_ESM.docx]

**Supplemental Information**

**Barker Model**

Cormac-Jolly-Seber (CJS) models ([Cormack 1964](#_ENREF_6); [Jolly 1965](#_ENREF_7); [Seber 1965](#_ENREF_11)), which are a class of capture-mark-recapture (CMR) models, have been well tested and used for survival estimation of marked fish in large ([Burnham et al. 1987](#_ENREF_4)) and small freshwater tributaries. However, CJS models require captures and recaptures (active sampling period) to occur during discrete and short, relative to the period between active sampling occasions, sampling occasions. Data from passive instream antennas (PIA), which collect data continuously on passive integrated transponder (PIT) marked individuals, does not naturally fit within the CJS sampling framework. More recently, CMR models, such as the Barker model ([Barker 1997](#_ENREF_1)), have been developed to accommodate continuously collected resight and recovery data between sampling occasions. Sandercock ([2006](#_ENREF_10)) provides a good comparison of the CJS and Barker models in his review of CMR models.

Because the majority of survival data was continuously collected by PIAs, we used the Barker model ([Barker 1997](#_ENREF_1)) rather than a CJS model to estimate survival. The Barker model is a re-parameterization of the CJS model includes continuously collected resight and recovery data between sampling occasions in the likelihood function ([Barker et al. 2004](#_ENREF_2)). There are 7 parameters in the Barker model ([from Cooch and White 2016](#_ENREF_5)): Si = the probability an animal alive at i is alive at i +1, pi = the probability an animal at risk of capture at i is captured at i, ri = the probability an animal that dies in interval i to i +1 is found dead, Ri = the probability an animal that survives from i to i + 1 is resighted (alive) sometime between i and i +1, R′i = the probability an animal that dies in i, i +1 without being found dead is resighted alive in i, i + 1 before it died, Fi = the probability an animal at risk of capture (i.e., on study area) at i is at risk of capture at i +1, and F′i = the probability an animal not at risk of capture at i is at risk of capture at i + 1 (this differs from the definition in Barker 1997).

**Model Construction**

We constructed different encounter history input files for fish in the treatment (Bridge Creek) and control (Murderers Creek) watersheds. We followed the same model construction and selection procedures for both data sets. To minimize the number of models assessed simultaneously and avoid false significance issues ([Burnham and Anderson 2002](#_ENREF_3)), our model development procedure was a sequential process ([Nichols et al. 1997](#_ENREF_9)). We began by constructing a global model each parameter estimated for each time period (t; S(t) p(t) r(t) R(t) R’(t) F(t) F’(t)). Then, leaving survival with the global structure (i.e., S(t)), we constructed models with more parsimonious structures for the remainder of the parameters in which estimates were constant (‘.’ in model notation) across time periods (e.g., p(.), R(.), F(.), etc.). We also constructed models that fixed r = 0 (r(.=0) in model notation) and F’ = 0 because there were no dead recoveries during the study period and no juvenile steelhead were encountered returning to a watershed after leaving the downstream terminus (as indicated by the PIA there) because they were migrating to the ocean. We also tried fixing R’= 0 in the top models because most of the estimates of R’ were extremely low (<0.0001),

We evaluated model fit and estimated the overdispersion parameter ($\hat{c}$) using the median $\hat{c}$ procedure in Program MARK under the most global model that converged well ([Cooch and White 2016](#_ENREF_5)). Deviations of *ĉ* above 1.0 indicate overdispersion and a *ĉ* >2–3 often indicates a lack of fit for the model ([Lebreton et al. 1992](#_ENREF_8); [Cooch and White 2016](#_ENREF_5)). If *ĉ* was >1 and <2, we adjusted AIC*_c_* by *ĉ* (AIC*c*) for model selection and used *ĉ* to inflate variances of parameter estimates.

**Model Results**

We ran the median $\hat{c}$ procedure on the global model for Bridge Creek and the S(t) p(t) r(.=0) R(t) R'(t) F(t) F'(.=0) for Murderers Creek because the top model did not converge well. Median $\hat{c}$ was <1 for both data sets. This indicated that there was no lack of model fit, so we proceeded without adjusting for AIC*_c_* was used for model selection. The top model for the control watershed (Murders Creek) was less parameterized than the top model for the treatment watershed (Bridge Creek; Tables 1a and 1b). This is likely because the sample size (number of marked fish) was 2.94× greater for the treatment site (n = 13,620) than for the control sites (n = 4,637). The structure of both top models had r and F fixed equal to zero, reflecting the lack of dead recoveries or any fish moving back on to the study watersheds after they were detected leaving at the downstream PIA at the mouth of the tributary. The top model for Murders was identical to the second best model except F’ was fixed to 0; the estimates of survival were the same to the third decimal place. Thus, both data sets had a clear top model, which was used in subsequent MCMC simulations.

Table 1a. Barker survival models for juvenile steelhead captured and marked in Bridge Creek, Oregon, June 2007– September 2012. Models shown are ordered by AIC*_c_*.

| Model^a^ | AICc | ΔAICc | wi | K | Deviance | Model Liklihood |
| --- | --- | --- | --- | --- | --- | --- |
| S(t) p(t) r(.=0) R(t) R'(t) F(t) F'(.=0) | 33,807.41 | - | 0.73 | 76 | 33,654.65 | 1.00 |
| S(t) p(t) r(.=0) R(t) R'(t) F(t) F'(.) | 33,809.43 | 2.02 | 0.27 | 77 | 33,654.65 | 0.36 |
| S(t) p(t) r(t) R(t) R'(t) F(t) F'(t) | 34,111.19 | 303.78 | - | 104 | 33,901.78 | 0.00 |
| S(t) p(t) r(.=0) R(t) R'(.=0) F(t) F'(.=0) | 34,148.25 | 340.84 | - | 62 | 34,023.75 | 0.00 |
| S(t) p(t) r(.=0) R(t) R'(.) F(t) F'(.=0) | 34,150.27 | 342.86 | - | 63 | 34,023.75 | 0.00 |
| S(t) p(t) r(.=0) R(t) R'(.=0) F(.) F'(.=0) | 34,191.35 | 383.95 | - | 48 | 34,095.05 | 0.00 |
| S(t) p(.) r(.=0) R(t) R'(.) F(t) F'(.=0) | 34,208.16 | 400.75 | - | 49 | 34,109.84 | 0.00 |
| S(t) p(t) r(.=0) R(.) R'(.) F(t) F'(.=0) | 36,235.13 | 2,427.72 | - | 49 | 36,136.82 | 0.00 |
| S(t) p(.) r(.=0) R(.) R'(.) F(.) F'(.=0) | 36,605.40 | 2,797.99 | - | 20 | 36,565.34 | 0.00 |
| S(t) p(.) r(.) R(.) R'(.) F(.) F'(.) | 36,609.41 | 2,802.00 | - | 22 | 36,565.34 | 0.00 |
| S(.) p(.) r(.) R(.) R'(.) F(.) F'(.) | 38,155.89 | 4,348.48 | - | 7 | 38,141.88 | 0.00 |

Table 1b. Barker survival models for juvenile steelhead captured and marked in Murderers Creek, Oregon, June 2007– September 2012. Models shown are ordered by AIC*_c_*.

| Model^a^ | AICc | ΔAICc | wi | K | Deviance | Model Liklihood |
| --- | --- | --- | --- | --- | --- | --- |
| S(t) p(t) r(.=0) R(t) R'(.=0) F(.) F'(.=0) | 9,910.13 | - | 0.98 | 47 | 9,815.21 | 1.00 |
| S(t) p(t) r(.=0) R(t) R'(.=0) F(t) F'(.=0) | 9,918.84 | 8.71 | 0.01 | 60 | 9,797.34 | 0.01 |
| S(t) p(t) r(.=0) R(t) R'(.) F(t) F'(.=0) | 9,920.89 | 10.76 | 0.00 | 61 | 9,797.34 | 0.00 |
| S(t) p(.) r(.=0) R(t) R'(.) F(t) F'(.=0) | 9,924.82 | 14.69 | 0.00 | 47 | 9,829.90 | 0.00 |
| S(t) p(t) r(.=0) R(t) R'(t) F(t) F'(.=0) | 9,950.84 | 40.71 | - | 76 | 9,796.43 | 0.00 |
| S(t) p(t) r(.=0) R(t) R'(t) F(t) F'(.) | 9,959.71 | 49.57 | - | 77 | 9,803.23 | 0.00 |
| S(t) p(t) r(t) R(t) R'(t) F(t) F'(t) | 10,049.70 | 139.57 | - | 104 | 9,837.17 | 0.00 |
| S(t) p(t) r(.=0) R(.) R'(.) F(t) F'(.=0) | 10,561.99 | 651.86 | - | 47 | 10,467.07 | 0.00 |
| S(t) p(.) r(.=0) R(.) R'(.) F(t) F'(.=0) | 10,618.57 | 708.44 | - | 36 | 10,546.03 | 0.00 |
| S(t) p(.) r(.=0) R(.) R'(.) F(.) F'(.=0) | 10,773.65 | 863.52 | - | 22 | 10,729.44 | 0.00 |
| S(.) p(.) r(.) R(.) R'(.) F(.) F'(.) | 11,114.48 | ###### | - | 7 | 11,100.45 | 0.00 |

^a^ Key to model notation: K = no. of parameters ; AICc = Akaike Information Criteria corrected for small sample size; ΔAICc = difference between the model listed and the AICc of the best model; wi = model weight based on model AICc compared to all other model AICc values. Lower case t indicates estimates are categorical by season (15 seasons), the period symbol indicates no difference across time, and .= 0 indicates a parameter set to 0. See **Barker Model** section above for a description of model parameters.

**References**

Barker, R. J. (1997). Joint modeling of live-recapture, tag-resight, and tag-recovery data. *Biometrics, 53*(2), 666-677.

Barker, R. J., Burnham, K. P., & White, G. C. (2004). Encounter history modeling of joing mark-recapture, tag-resighting and tag-recovery data under temporary emigration. *Statistica Sinica, 14*, 1037-1055.

Burnham, K. P., & Anderson, D. R. (2002). *Model selection and multimodel inference: Second Edition*. New York, New York, USA: Springer-Verlag.

Burnham, K. P., Anderson, D. R., White, G. C., Brownie, C., & Pollock, K. H. (1987). *Design and analysis methods of fish survival experiments based on release-recapture. Monograph 5* (Vol. Monograph 5, Vol. Monograph 5). Bethesda, Maryland, USA: American Fisheries Society.

Cooch, E. G., & White, G. C. (2016). Program MARK: "A gentle introduction", 14th Edition. Available at <http://www.phidot.org/software/mark/docs/book/>.

Cormack, R. M. (1964). Estimates of survival from the sighting of marked animals. *Biometrika, 51*, 429-438.

Jolly, G. M. (1965). Explicit estimates from capture-recapture data with both death and immigration-stochastic model. *Biometrika, 52*, 225-247.

Lebreton, J.-D., Burnham, K. P., Clobert, J., & Anderson, D. R. (1992). Modeling survival and testing biological hypotheses using marked animals: a unified approach with case studies. *Ecological Monographs, 62*(1), 67-118.

Nichols, J. D., Hines, J. E., & Blums, P. (1997). Tests for senescent decline in annual survival probabilities of common pochards, *Aythya ferina*. *Ecology, 78*, 1009-1018.

Sandercock, B. K. (2006). Estimation of Demographic Parameters from Live-Encounter Data: a Summary Review. *Journal of Widlife Management, 70*(6), 1504-1520, doi:doi:10.2193/0022-541X(2006)70[1504:EODPFL]2.0.CO;2.

Seber, G. A. F. (1965). A note on the multiple recapture census. *Biometrika, 52*, 249-259.
